# Planful Self-Control, Metabolic Risk, and Psychosocial Outcomes Among Young, Black Men: A Test of Skin-Deep Resilience Theory

**DOI:** 10.3389/fpsyg.2022.806955

**Published:** 2022-06-09

**Authors:** Steven M. Kogan, Ava J. Reck, Michael G. Curtis, Heather Zuercher, Christopher Collins, Elizabeth Kwon, Danielle A. Augustine

**Affiliations:** ^1^Center for Family Research, Owens Institute for Behavioral Research, University of Georgia, Athens, GA, United States; ^2^Department of Human Development and Family Science, University of Georgia, Athens, GA, United States; ^3^Carl Vinson Institute of Government, University of Georgia, Athens, GA, United States

**Keywords:** metabolic risk, skin-deep resilience, black men, self-control, childhood adversities

## Abstract

Research on skin-deep resilience suggests that for youth and young adults from disadvantaged backgrounds, high levels of planful self-control may promote positive psychosocial outcomes while simultaneously conferring vulnerabilities to chronic diseases related to aging. In this study, we investigated the divergent effects of planful self-control on young Black American men’s psychosocial well-being and their metabolic risk. We expected that high levels of planful self-control in emerging adulthood would predict positive outcomes in young adulthood (educational attainment, low depressive symptoms, job satisfaction); however, the combination of high levels of planful self-control and the experience of contextual adversity either in emerging adulthood or in childhood would forecast poor metabolic health. Hypotheses were tested with prospective data from 504 Black American men followed from age 20 to age 26. Planful self-control in emerging adulthood directly forecasted low levels of depressive symptoms, one’s likelihood of obtaining a bachelor’s degree, increased job satisfaction, and increases in metabolic risk. Exposure to childhood deprivation moderated the influence of planful self-control on metabolic risk. Men with high levels of deprivation and high levels of planful self-control exhibited the worst metabolic profiles in the sample. In contrast, men with high levels of childhood deprivation and low levels of planful self-control exhibited the best metabolic profiles. Documenting the health consequences associated with planful self-control provides a foundation from which to identify modifiable psychosocial factors that affect the course of psychosocial problems and health.

## Introduction

Residence in the rural South of the US takes a toll on Black men’s health (Quarells et al., 2012). Research indicates that their life expectancy averages 67.7 years, more than 6 years less than rural white men (Thorpe et al., 2013; Singh and Siahpush, 2014). Although accidents and violence play an important and tragic role in reducing life expectancy, particularly among younger men, much of this disparity is a consequence of morbidity and mortality related to chronic diseases of aging (Gilbert et al., 2016; Pathak, 2018). For example, compared with Caucasians, Black men evince earlier onset and greater prevalence of, and greater mortality from, coronary heart disease (CHD; Graham, 2015). They are twice as likely to develop type 2 diabetes and are more extensively affected by its complications, including CHD, blindness, and amputation (Mainous et al., 2004). These chronic health problems can take decades to develop and to begin to manifest clinically. However, risk for and resilience to these health problems can be studied earlier in development by tracking intermediate biological processes that are known to contribute to disease progression. Specifically, studies have examined markers of future cardiovascular and metabolic health problems, including elevated blood pressure, lipids, insulin resistance, and obesity (Cornier et al., 2008). We focus in this study on health risks among young adult Black men.

For Black Americans in general, and Black men in particular, life in rural areas can be more challenging than in other areas due to restricted educational and employment opportunities, barriers to physical and mental health care, and a lack of public transportation (Probst and Fozia, 2019). The challenge of overcoming the environmental obstacles associated with chronic economic stress is exacerbated by institutional and interpersonal experiences with racial discrimination (Kogan and Bae, 2020). Stressful experiences proliferate in high poverty environments and during childhood, rural Black men experience elevated rates of adverse childhood experiences associated with poverty (National Advisory Committee on Rural Health and Human Services, 2018). The challenges continue as men transition to the labor force. Although approximately 50% pursue postsecondary education or training programs, completion rates are low: fewer than half of the men who enroll will complete postsecondary training programs or obtain a university degree (Shapiro et al., 2017). Due to poor preparation in schools and discriminatory hiring practices, most young men obtain part- or full-time employment in low-wage jobs that offer little training and no opportunity for advancement (Bucknor, 2015). Job turnover rates are high, and prolonged periods of unemployment grow increasingly common. For men with little stake in conventional educational or occupational systems, the transition to independent adult roles can be a demoralizing and protracted process that taxes their self-efficacy.

Despite the challenges associated with living in a low-resource rural environment, many rural Black American men are able to protect themselves from negative effects of their environment (Brown, 2008; Zapolski et al., 2014; Kogan and Bae, 2020). These men have been called *resilient* because their competence develops in the face of the contextual adversity with which they must contend, enabling them to adapt and “beat the odds” that their lives have presented to them (Teti et al., 2012; Denckla et al., 2020). Research suggests that high levels of *planful self*-*control* may be critical to resilient outcomes. Planful self-control is a group of attributes involved in the self-regulation of cognition, emotion, and behavior (de Ridder et al., 2012). It involves planning, persistence, and a future goal orientation which supports academic and vocational achievement, and psychological adjustment despite a lifetime of exposure to the barrier’s endemic to the rural South (Brody et al., 2020). Common outcomes of high levels of planful self-control include lower rates of depression, and high educational and vocational involvement (Spencer, 2001; Silverstein et al., 2021).

Recent research focused on rural Black Americans has documented a paradoxical effect whereby youths from low-income families who are exhibiting high levels of planful self-control and striving hard to succeed experience good mental health but are at elevated risk for adverse health outcomes. These studies find that higher planful self-control during childhood among youths growing up in either economically disadvantaged families or impoverished neighborhoods is associated with better psychosocial outcomes, as reflected in less drug use, lower levels of depressive symptoms, and college attendance. Examination of health vulnerabilities, however, reveals that high levels of planful self-control are associated with higher allostatic load scores (Brody et al., 2013; Chen et al., 2015), a metric that reflects future health vulnerability. The diverging effects of planful self-control have been termed, “skin-deep resilience,” wherein outward indicators of competence co-occur with poor health in upwardly mobile Black young people exposed to social adversity.

Skin-deep resilience research suggests that the stress of surmounting adversity to achieve positive developmental outcomes, such as greater educational and vocational success, may tax physiological systems that respond to stress, including the sympathetic nervous system (SNS), and the hypothalamic–pituitary–adrenal (HPA) axis (Merritt et al., 2011). Young Black men exposed to social adversity develop a range of strategies to cope with chronic and acute stressors (Ellis et al., 2015). Some men will use maladaptive coping strategies such as anger, resignation, or substance use. These coping strategies can promote a range of negative psychosocial outcomes including depression, limited educational attainment, and vocational distress. In contrast, some young people who encounter chronic challenges “put their heads down” and persist in pursuit of life goals with even greater determination to succeed. This high effort coping style that promotes success in many life pursuits and deters depressive symptoms appears to tax the physiological systems that respond to stress, leading to a greater risk of elevations in markers for metabolic problems (DeAngelis, 2020).

In this study, we investigated the divergent effects of planful self-control on young adult, Black American men’s psychosocial well-being and their metabolic risk. To advance understandings regarding the skin-deep resilience phenomenon, we investigated hypotheses related to the timing and type of social adversity men have experienced. Several studies examining the link between adversity and factors associated with dysregulation in the HPA axis and sympathetic nervous system suggest that childhood exposures to stress have life-course-persistent effects (Miller et al., 2011; Evans et al., 2012). Even if an individual exposed to adversity as a child experience a more optimal environment as a young adult, the physiological “residue” of childhood adversity remains and manifests as increased allostatic load, metabolic risk, and systemic inflammation (Miller et al., 2011; Evans et al., 2012). We thus examine separately the potential influences of adversity experienced during childhood and adolescence vs. stressors experienced during emerging adulthood.

Recent research also indicates the importance of considering distinct dimensions of childhood adversity. McLaughlin et al. (2014) highlighted two dimensions: the absence of cognitive and social stimulation, termed *deprivation*, and the presence of experiences involving harm or risk thereof, termed *threat*. Unique emotional, cognitive, and neurobiological pathways have been proposed that underlie the association of these dimensions of adversity with downstream outcomes (McLaughlin et al., 2014). Deprivation affects mental health via influences on the development of higher-order cognitive processes, such as linguistic processing and executive function. Threat in childhood affects mechanisms involved in the acquisition and extinction of fear, with downstream consequences on emotion processing. The extent to which deprivation or threat modulates the effect of planful self-control on metabolic risk has not been investigated.

**FIGURE 1:**
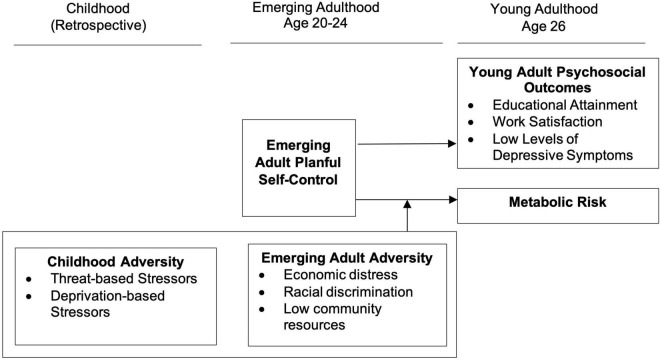
Conceptual model of study hypotheses.

Figure 1 presents a summary of the skin-deep resilience hypotheses we investigated. We first examined the direct influence of planful self-control on three psychosocial outcomes: educational attainment, vocational satisfaction, and depressive symptoms. Consistent with past research on similar outcomes with youth and young adults (Brody et al., 2013, 2016, 2020; Chen et al., 2020), we expect planful self-control to forecast greater educational attainment and vocational success, along with low levels of depressive symptoms. In contrast, we expect the effects of planful self-control on metabolic risk to depend on men’s exposure to social adversity including exposure to deprivation and threat during childhood, and contextual stressors during emerging adulthood. Specifically, in the context of high levels of social adversity, planful self-control in emerging adulthood will forecast worse metabolic outcomes in young adulthood.

## Methods and Measures

### Participants

We tested study hypotheses with data from 504 Black American men recruited from 11 rural counties in South Georgia. Eligibility criteria included self-identifying as Black or African American and male, residing in the sampling area, and being between 19 and 22 years of age (M_age_ = 20.29; SD_age_ = 1.10) at baseline (Time 1 [T1]). We recruited participants using respondent-driven sampling (Heckathorn, 2002), an effective method for recruiting hard-to-reach samples of young adults (Kogan et al., 2011). Emerging adult rural Black men are a hard-to-reach population due to frequent transitions in living arrangements, changing contact information, and the lack of reliable lists from which to randomly sample. Community liaisons recruited 45 initial participants from 11 contiguous, rural counties in South Georgia, representing areas of rural poverty in the Southeastern U.S. (Probst and Fozia, 2019). These initial participants were members of the community liaisons’ social networks, or individuals who responded to advertisements and outreach in the community. The 45 initial participants completed baseline surveys and were then asked to identify three other Black American men in their community that qualified for the study. Project staff then contacted the referred participants. After the referred participants completed the survey, they were asked to refer three more men from their networks. Each referring participant received $25 per person who completed the survey. This continued until the sampling goal (*N* ≥ 500 per *a priori* power analysis) was reached.

Respondent-driven sampling methods compensate for the non-random recruitment of the sample (Heckathorn, 2002). Analyses of network data at baseline suggested that the sample evinced negligible levels of bias arising from the characteristics of the initial participants, recruitment efficacy, and differences in the sizes of participants’ networks (Kogan et al., 2016). Thus, we used raw data in the current analysis.

### Procedures

Participants completed surveys in their homes or at a convenient location in the community. Research staff administered surveys on a laptop computer using an audio computer-assisted self-interview protocol, which provides survey navigation with voice and video enhancements to alleviate literacy concerns. Participants were compensated $100 for completing the survey. Participants provided written informed consent; all study protocols were approved by the University’s Human Subjects Review Board. The study operated under a federal certificate of confidentiality issued by the U.S. National Institute of Health.

**FIGURE 2:**
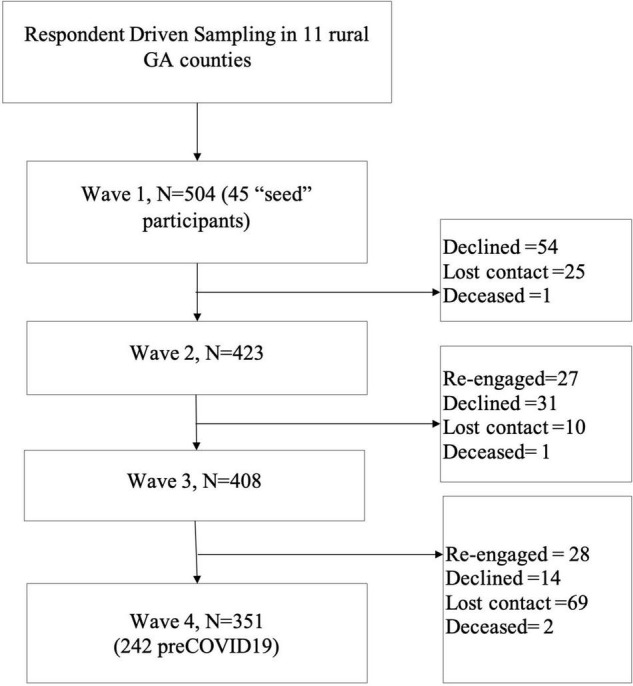
Participant retention.

Men provided survey data at three additional waves (T2, T3, and T4; see Figure 2). Data from waves T1, T2, and T3 were collected during men’s emerging adult years. Baseline data were collected between January 2012 and August 2013 when participating men’s mean age was 20.26 (SD = 1.08). A follow-up survey at T2 was conducted in the same manner between August 2013 and March 2015. The T3 data collection took place between April 2015 and December 2016 when participants’ mean age was 23.11 (SD = 1.25). The young adult data collection (T4) was conducted between March 2019 and March 2021, when the mean age of the sample was 27.67 (SD = 1.39). At T4, about 70% (*n* = 351) of participants remained in the study.

At T4, blood spot specimens were collected; research staff conducted a finger prick and helped the participant deposit blood spots on data collection cards. Specimen cards were sent to ZRT Laboratories (Beaverton, OR) where levels of cholesterol, triglycerides, and glycosylated hemoglobin (HbA1c) were assayed. Research staff collected participant’s blood pressure using an Omeron automatic blood pressure cuff. The research staff also measured the participants’ waist circumference at the midpoint between the upper iliac crest and lower costal margin. All anthropomorphic measurements were taken twice and subsequently averaged.

#### COVID and Study Protocols

In March 2020, when T4 data collection for 242 participants was completed, COVID-19 related lockdowns and in-person visit restrictions were imposed. This resulted in a 2-month pause in research visits when we could only collect data remotely (*N* = 21). Participants were sent a link to the study survey and no biomarkers were measured. In October 2020, when data collection resumed, study procedures had to be adapted to provide for additional COVID-19 protections for both study participants and research staff. The adapted procedures entailed limiting close contact with participants to 15 min or less. Thus, for the survey part of the data, participants were sent a survey link and they completed the survey on their own devices. Research assistants then delivered a kit with data collection instructions to each participant’s home. Study staffs were available by video chat for assistance, and participants were instructed on how to measure their own height, weight, waist and hip circumference, and blood pressure. Then, blood samples were collected outside the home by trained research staff using the same procedures as pre-COVID. Of 351 men who participated at T4, 242 participated before COVID restrictions were imposed, with 177 of them providing blood sample (73.14%); After COVID restrictions were imposed, 109 men participated in the study with 69 of them (63.3%) submitting blood samples through contact-limited procedures.

We ran independent sample *T*-tests to see if study variables were significantly different between data collected pre-COVID and post-COVID. *T*-tests revealed that data collected pre- and post-COVID did not differ significantly for planful self-control (*p* = 0.21), childhood threat (*p* = 0.90), childhood deprivation (*p* = 0.80), or contextual stress (*p* = 0.45). *T*-tests revealed a significant difference in metabolic risk between participants whose data were collected pre-COVID vs. post-COVID (*p* = 0.02). Thus, COVID-19 data collection timing was controlled in all analyses. We conducted a second independent sample *T*-test to compare planful self-control, childhood threat, childhood deprivation, and contextual stress between the participants who submitted biodata compared to the participants who completed the survey only. *T*-tests suggest in-person visits, compared with survey-only data collection, did not differ significantly for planful self-control (*p* = 0.40), childhood threat (*p* = 0.32), childhood deprivation (*p* = 0.64), or contextual stress (*p* = 0.94).

### Measures

#### Metabolic Risk

Bloodspots and anthropometric protocols yielded assessments of central adiposity, high-density lipoprotein (HDL), cholesterol, triglycerides, HbA1c, and blood pressure. We made an index variable summing the number of health risk factors at T4. Following previous studies (Han et al., 1995; Expert Panel on Detection, 2001), we dichotomized measures so that they indicate presence (1) or absence (0) of risk per International Diabetes Federation (IDF) criteria (Alberti et al., 2009). Central adiposity was coded as 0 (waist circumference < 94 cm) or 1 (waist circumference ≥ 94 cm). HDL was coded as 0 (>40 mg/dL) or 1 (≤40 mg/dL). Triglycerides were coded as 0 (<150 mg/dL) or 1 (≥150 mg/dL). HbA1c was coded as 0 (≤7) or 1 (>7). Elevated blood pressure was coded as 1 when systolic pressure was above or equal to 130 millimeters of mercury (mm Hg) and/or diastolic blood pressure was at or above 85 mm Hg, or 0 if systolic blood pressure was below 130 mm Hg and/or diastolic blood pressure was below 85 mm Hg. The resulting metabolic risk index ranged from 0 to 5 (*M* = 1.38, SD = 1.09).

#### Planful Self Control

Planful self-control was measured at T1, T2, and T3 using three measures. Self-regulation was measured using a 10-item version of the Self-Regulation Questionnaire (Brown et al., 1999). The response scale ranged from 1 (*strongly disagree*) to 4 (*strongly agree*). Example items included, “If I wanted to change, I am confident that I could do it,” and “I usually keep track of my progress toward my goals.” Cronbach’s α was as follows: T1 α = 0.90, T2 α = 0.95, T3 α = 0.96. Self-regulation scales across time were correlated (*r*s > 0.54, *p* < 0.00) and subsequently averaged. State hope was measured using the 6-item State Hope Scale (Snyder et al., 1996). The response scale ranged from 1 (*strongly disagree*) to 4 (*strongly agree*). Example items included, “I can think of many ways to reach my current goals,” and “Right now, I see myself as being pretty successful.” Cronbach’s α was 0.85 at T1, 0.90 at T2, and 0.93 at T4, and the scores across time were correlated (*r*s > 0.25, *p* < 0.00). They were averaged to form a composite. Men completed the nine-item Perceived Life Chances scale (Jessor et al., 1998). The scale provided a list of positive life experiences and asked men how sure they were that they would achieve them. The response scale ranged from 1 (*not sure at all*) to 4 (*very sure*). Example items included, “You will have a job that pays you well,” and “You will be respected in your community.” Alphas were as follows: T1 α = 0.90, T2 α = 0.92, T3 α = 0.95, and the scores across time were correlated (*r*s > 0.32, *p* < 0.00). To provide a reliable index of emerging adult planful self-regulation, the scales were averaged across T1, T2, and T3. The resulting three composites were all significantly correlated at or above 0.50, standardized and summed to create a T1–T3 planful self-control composite measure.

#### Childhood Threat

At T1, men reported on childhood experiences of threat using eight-items from the Adverse Childhood Experience Questionnaire (Felitti et al., 1998). The scale lists a range of experiences involving physical abuse, sexual abuse, and witnessing domestic violence and asks men if the experiences occurred to them in their first 16 years of life. The response scale was 1 (*yes*) or 0 (*no*). Example items include “Did a parent or other adult hit you do hard that you had marks or were injured?,” “Did an adult or person at least 5 years older than you ever attempt or actually have oral, anal, or vaginal intercourse with you” and “Was your mother or stepmother often pushed, grabbed, slapped, or had something thrown at her?” Items were summed to create a childhood threat scale that ranged from 0 to 8. Cronbach’s α was 0.80.

#### Childhood Deprivation

Childhood deprivation was assessed with measures of neglect and childhood poverty at T1. Neglect was measured using four items from the Adverse Childhood Experience Questionnaire (Felitti et al., 1998). Men were asked if a list of experiences occurred to them within their first 16 years of life, including, “you didn’t have enough to eat, had to wear dirty clothes, and had no one to protect you?” and “your parents were too drunk or high to take care of you or take you to the doctor if you needed it?” Cronbach’s α was 0.70. Childhood family poverty was measured with a 15-item scale (Curtis et al., 2021). The scale asked men how often they experienced poverty-related events in their first 16 years of life. Example items include, “My family often had to move due to money problems” and “I often did not have clothes that fit.” The response scale ranged from 0 (*never*) to 3 (*almost always*). Cronbach’s α was 0.81. The neglect and poverty scales were significantly correlated (*r* = 0.30). They were standardized and summed to create a childhood deprivation measure.

#### Emerging Adult Contextual Stress

We constructed a contextual stress index using three indicators (economic distress, racial discrimination, and low community resources) assessed at T1, T2, and T3. Men reported on their experience of economic distress in the past 6 months with five statements about their economic resources such as, “I have enough money to afford the kind of food I need,” and “I have enough money to afford the kind of medical care I need.” The men’s responses ranged from 1 (*strongly disagree*) to 4 (*strongly agree*). All items were reverse-scored and summed to create a total economic stress variable. Cronbach’s α was as follows: T1 = 0.79, T2 = 0.84, T3 = 0.87. Racial discrimination was assessed using the nine-item Racist Hassles Questionnaire (Brody et al., 2006). Men’s responded to the items on a scale ranging from 0 (*never*) to 3 (*frequently*). Cronbach’s α was as follows: T1 = 0.84, T2 = 0.86, T3 = 0.87. Examples of items include, “have you been ignored, overlooked, or not given service because of your race,” and “have you been called a name or harassed because of your race.” Men responded to the 12-item community resources subscale of the Community Resources and Problems Scale (Forehand et al., 2000). Men were asked how well their community was at providing a list of resources including “finding full-time jobs,” “public transportation” and “finding places to live on your own.” The response scale ranged from 1 (*very poor*) to 5 (*very good*). All items were reversed scored and summed, so higher scores reflected lower community resources. Cronbach’s α was as follows: T1 = 0.94, T2 = 0.96, T3 = 0.94. All measures were significantly correlated (rs > 0.25) and were standardized and summed to create the contextual stress variable.

#### Depressive Symptoms

At T4, men responded to the 20-item Center for Epidemiological Studies Depression scale (Radloff, 1977). The scale asks participants to rate how often they experienced a range of depressive symptoms over the previous week. Example items include, “How often were you bothered by things that usually do not bother you” and “How often did you feel sad?” The response scale ranged from 0 (*rarely or none of the time*) to 4 (*most or all of the time*). Items were summed to create a total depressive symptom scale that ranged from 0 to 60. Cronbach’s alpha was 0.86 at T4.

#### Job Satisfaction

Job satisfaction was measured at T4 using an 18-item scale (Gattiker and Larwood, 1986). The scale asked men who reported that they worked full or part-time (*n* = 279) if they agreed or disagreed with a list of statements about their job, such as “I am in a position to do mostly work which I really like” and “I am receiving fair compensation compared to my peers.” The response scale ranged from 1 (*strongly disagree*) to 4 (*strongly agree*). Cronbach’s alpha was 0.88.

#### Educational Attainment

At T4 men reported their educational attainment on a 6-point scale ranging from 1 (10th grade or less) to completing 6 (*receipt of a graduate degree*). Educational attainment was recoded as 0 (*no bachelor’s degree*) or 1 (*bachelor’s degree or higher*).

#### Covariates

To isolate the individual and interaction effects of planful self-control and social adversity on Black men’s metabolic risk in emerging adulthood, we controlled for several pre-existing factors that may be associated with metabolic risk. At T1, Men reported on their mother and father’s highest level of education. Responses ranged from 1 (*10th grade or less*) to 6 (*receipt of a graduate degree*). At T4 men reported on the number of hours they spent doing moderate or vigorous exercise in the previous week. Physical activity was coded as the average number of hours the participant engaged in moderate and/or vigorous exercise per week. The final psychical activity score ranged from 0 to 28 h (*M* = 6.58, SD = 7.9).

Men reported on their alcohol and tobacco use at T4. Men were asked how many cigarettes or cigars they had smoked in the previous 3 months. The response scale ranged from 0 (*none at all*) to 7 (*more than 40 per day; M* = 3.08, *SD* = 4.37). Men were then asked on average, how many alcoholic drinks they consume each month (*M* = 4.27, SD = 5.77).

Men reported on their consumption of a healthy diet at T4 using the 23-item Food Habits Checklist (Johnson et al., 2002). The checklist asked men about their diet choices with a response option of 1 (*true*) or 0 (*false*). Example items include, “I try to keep my overall fat intake down,” “I make sure I eat at least one serving of vegetables or salad a day,” and “I rarely eat fast food meals.” Items were summed to create a healthy diet scale that ranged from 0 to 23. Cronbach’s alpha was 0.70.

Age was measured at T1 via birth dates (*M* = 20.1, SD = 1.27). COVID-19 occurred during the fourth wave of data collection in the current study. We created a dummy variable that indicated whether the data had been collected prior to the first national lockdown on March 11th, 2020. The variable was coded as 0 (*prior to March 11th, 2020*) and 1 (*post-March 11th, 2020*). Data collection timing vis-á-vis COVID-19 was controlled in all analyses.

### Data Analysis

Tests of study hypotheses were conducted using Mplus 8 (Muthén and Muthén, 1998-2017). Little’s Missing Completely at Random test indicated that our data were missing completely at random for all study variables [χ^2^_(679)_ = 614.63, *p* = 0.96]. Accordingly, missing data were managed with full information maximum likelihood (FIML) estimation (Little and Rubin, 2019). FIML tests hypotheses with all available data; therefore, no cases were dropped due to missing data (Arbuckle, 1996; Little and Rubin, 2019). Data from all 504 participants were included in tests of study hypotheses.

Analyses were conducted in stages. First, we assessed the impact of planful self-control on each psychosocial outcome (depressive symptoms, job satisfaction, educational attainment). Linear regression analyses were used to examine the effects on continuous outcomes (depressive symptoms and job satisfaction), and logistic regression was used to analyze the effects on the dichotomous outcome (educational attainment). In all analyses childhood threat, childhood deprivation, emerging adult contextual stress, age, parental education, and COVID-19 data collection were controlled. Comparative Fit Index (CFI) values greater than 0.95, Root Mean Square Error of Approximation (RMSEA) values less than 0.08, and a χ^2^/*df* ratio less than 3.0 indicate acceptable model fit (Hu and Bentler, 1999). Educational attainment was a dichotomous outcome, and therefore a logistic regression was used.

Next, we conducted interaction analyses to test our hypotheses regarding the moderating influence of, threat, deprivation, and contextual stress on the association between planful self-control and metabolic risk. We used ordinal regression analysis to model the main effects of planful self-control behavior on metabolic risk while controlling for diet, physical activity, age, and COVID-19 data collection. To test our hypotheses regarding the moderating effects of childhood threat, deprivation, and contextual stress on the association between planful self-control and metabolic risk, we created interaction terms for each moderator and conducted hierarchical ordinal regressions (Marsh et al., 2013). Models with significant interaction effects were plotted using the Johnson-Neyman plot in Mplus (Johnson and Neyman, 1936; Muthén and Muthén, 1998-2017).

## Results

Table 1 presents the means, standard deviations, and frequencies among study variables. At T4, men were, on average, 27 years old (SD = 1.23). Men in the study had an average of 1.38 metabolic risk factors (SD = 1.09). Approximately 13.5% (*n* = 68) of men had 0 metabolic risk factors, 38.6% (*n* = 120) had 1 risk factor, 24.1% (*n* = 75) had 2 risk factors, and 15.4% had 3 or more risk factors (*n* = 48). Despite 72% of participants being employed (86% full time, 14% part-time), the average monthly income among working men was $871. Correlations of all study variables can be found in Supplementary Table 1.

**TABLE 1:** Means, standard deviations, and frequencies of study variables.

	Mean/n*	SD/%*
Metabolic risk index (0–5)	1.38	1.09
Central adiposity (waist circumference ≥94 cm)	107*	35.00*
High-density lipoprotein cholesterol (≤40 mg/dL)	121*	49.20*
Triglycerides (≥150 mg/dL).	69*	28.10*
HbA1c (>7)	7*	2.85*
Blood pressure (SBP ≥ 130 mm Hg and/or DBP ≥ 85 mm Hg)	156*	63.41*
Planful self-control	0	0.86
Childhood threat	1.31	1.87
Childhood deprivation	0	1.45
Contextual stress	0	2.09
Depressive symptoms	4.05	4.25
Job satisfaction	54.50	8.65
Educational attainment (≥Bachelor’s degree)	55*	15.67*
Age in years	20.10	1.27
Healthy diet	9.10	4.76
Physical exercise (hours/week)	13.16	4.33
COVID onset (data collected post COVID onset)	109*	31.05*
Maternal education	3.89	6.12
Paternal education	4.10	6.16
Tobacco use	3.08	4.37
Alcohol use (drinks/month)	4.26	5.77

SD, standard deviation. *In the left column signifies total number at the corresponding cut-off; *In the right column signifies the percent at the corresponding cut-off. HbA1c, hemoglobin A1c; SBP, systolic blood pressure; DBP, diastolic blood pressure.

Table 2 presents the results and model fit indices for the linear regression (depressive symptoms and job satisfaction) and logistic regression (educational attainment) analyses on psychosocial outcomes. Per Model 1, increased levels of planful self-control behaviors predicted reductions in depressive symptoms (β = −0.13, *p* = 0.03, 95% CI [−0.24, −0.01]). Per Model 2, planful self-control had a significant association with job satisfaction (β = 0.24, *p* < 0.01, 95% CI [0.12, 0.36]). Per Model 3 planful self-control was a significant predictor of educational attainment (β = 0.36, *p* < 0.01, 95% CI [0.17, 0.54]). Participants with more planful self-control behavior were 2.36 times more likely per unit change in the scale to graduate from college than those with less planful self-control.

**TABLE 2:** Effects of planful self-control, childhood threat, deprivation, and contextual stress on psychosocial outcomes.

	Depressive symptoms					Job satisfaction					Educational attainment				
	β	*p*	*b*	SE	95% CI	β	*p*	*b*	SE	95% CI	β	*p*	OR	SE	OR 95% CI
**Predictors**															
Planful self control	−0.13	0.03*	−0.77*	0.36	−1.47, −0.07	0.24***	0.00	2.41***	0.63	1.18, 3.64	0.36**	0.00	2.36	0.62	1.41, 3.94
Deprivation	0.07	0.27	0.24	0.22	−0.19, 0.67	−0.01	0.93	−0.03	0.38	−0.77, 0.71	−0.20	0.08	0.75	0.13	0.54, 1.05
Threat	0.06	0.34	0.15	0.16	−0.16, 0.46	0.03	0.66	0.13	0.29	−0.44, 0.69	0.16	0.07	1.20	0.12	0.98, 1.45
Contextual stress	0.18**	0.00	0.46**	0.14	0.19, 0.73	−0.21**	0.00	−0.87**	0.27	−1.40, −0.35	−0.02	0.78	0.98	0.07	0.85, 1.13
**Covariates**															
Maternal education	−0.18	0.42	−0.15	0.19	−0.52, 0.22	0.20	0.38	0.28	0.32	-0.35, 0.91	0.72	0.06	1.28	0.14	1.04, 1.57
Paternal education	0.15	0.50	0.13	0.19	−0.24, 0.50	−0.15	0.52	−0.21	0.32	-0.84, 0.42	-0.55	0.11	0.83	0.09	0.68, 1.02
Age	−0.08	0.16	−0.30	0.21	−0.71, 0.12	0.01	0.89	0.06	0.42	= 0.77, 0.88	−0.06	0.48	0.91	0.11	0.71, 1.18
COVID onset	−0.01	0.93	−0.05	0.65	−1.33, 1.22	−0.00	0.95	−0.08	1.26	−2.56, 2.40	−0.07	0.45	0.73	0.30	0.33, 1.65
**Model fit**															
χ^2^	16.60					16.65					−				
DF	12					12					−				
CFI	0.89					0.91					−				
RMSEA	0.02					0.03					−				

*Significant at the 0.05 level (2-tailed). **Significant at the 0.01 level (2-tailed). ***Significant at the 0.001 level (2-tailed). SE, standard error; 95% CI, unstandardized 95% confidence interval; OR, odd’s ratio; DF, degrees of freedom; CFI, comparative fit index; RMSEA, root mean square error of approximation.

**FIGURE 3:**
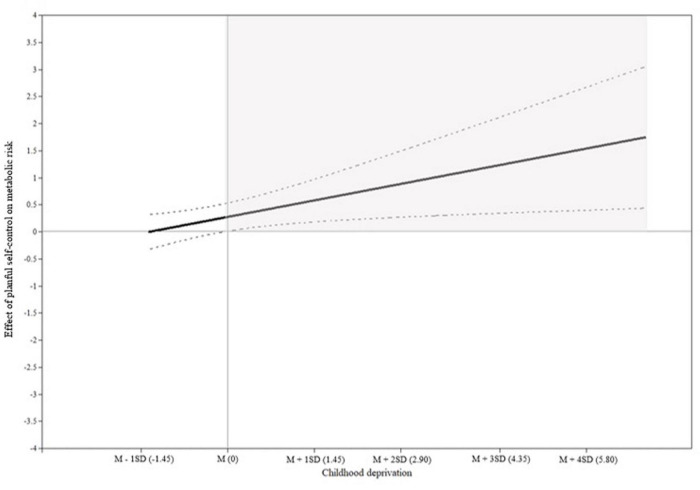
Johnson-Neyman plot. The gray shaded areas indicated the region of significance. The x-axis indicates the score of childhood deprivation and the y-axis represents the effects of planful self-control on metabolic risk. The black solid line represents the effects of planful self-control on metabolic risk corresponding to the values of childhood deprivation. Dashed lines show the 95% confidence interval.

Table 3 presents the results and model fit indices of the ordinal regression analyses on metabolic risk. Model 1 presents the main effects of planful self-control, childhood threat, childhood deprivation, contextual stress, and confounding variables on metabolic risk. Planful self-control was significantly associated with T4 metabolic risk (β = 0.12, *p* = 0.04). Participants with high planful self-control behaviors were 1.3 times more likely to have increased metabolic risk. Model 2 includes the interaction term (planful self-control × childhood deprivation) which was significant (β = 0.15, *p* = 0.02). Model 3 includes the interaction term between planful self-control and threat, which was non-significant (β = −0.01, *p* = 0.87). Model 4 includes the interaction term between planful self-control and contextual stress. This interaction was also non-significant (β = −0.06, *p* = 0.23). The Johnson-Neyman plot (see Figure 3) presents the effect of planful self-control on metabolic risk (y-axis) and the 95% confidence intervals across different values of childhood deprivation (x-axis). The shaded area on the right side of the plot indicates that for men who experienced higher levels of childhood deprivation, planful self-control is associated with elevated metabolic risk. This region of significance begins slightly below the mean, when childhood deprivation reaches -0.01, which comprises 34.1% of the sample.

**TABLE 3:** Effects of childhood threat, deprivation, and contextual stress by striving on metabolic risk.

	Model 1				Model 2				Model 3				Model 4			
	*b*	SE	Odds ratio	95% CI	*b*	SE	Odds ratio	95% CI	*b*	SE	Odds ratio	95% CI	*b*	SE	Odds ratio	95% CI
**Predictors**																
Planful self-control	0.27*	0.13	1.30	1.00, 1.69	0.28*	0.13	1.31	1.01, 1.71	0.27*	0.13	1.31	1.01, 1.69	0.26*	0.14	1.30	1.01, 1.69
Deprivation	−0.03	0.10	0.99	0.81, 1.20	−0.01	0.09	1.00	0.83, 1.20	−0.03	0.10	0.99	0.81 1.20	−0.02	0.10	0.98	0.80, 1.19
Threat	−0.08	0.08	0.92	0.79, 1.08	−0.07	0.08	0.93	0.80, 1.09	−0.08	0.09	0.92	0.78, 1.09	−0.09	0.08	0.92	0.78, 1.08
Contextual stress	0.04	0.05	1.04	0.93, 1.15	0.031	0.05	1.03	0.93, 1.14	0.04	0.06	1.04	0.93, 1.15	0.05	0.06	1.05	0.94, 1.17
**Interactions**																
Planful SC × Deprivation		-	-	-	0.22*	0.09	1.24	1.04, 1.49	-	-	-	-	-	-	-	-
Planful SC × Threat	-	-	-	-	-	-	-	-	−0.01	0.08	0.99	0.85, 1.14	-	-	-	-
Planful SC × Contextual stress	-	-		-	-	-	-	-	-	-	-	-	−0.06	0.05	0.94	0.86, 1.04
**Covariates**																
Healthy diet	0.00	0.03	1.00	0.96, 1.05	0.01	0.02	1.00	0.96, 1.05	0.01	0.03	1.00	0.96, 1.05	0.01	0.03	1.01	0.96, 1.06
Phys. exercise	0.00	0.01	1.00	0.99, 1.02	−0.00	0.01	1.00	0.98, 1.01	0.00	0.01	1.00	0.99, 1.02	0.00	0.01	1.00	0.99, 1.02
Age	0.07	0.09	1.07	0.90, 1.28	0.06	0.09	1.06	0.89, 1.26	0.08	0.09	1.06	0.89, 1.26	0.07	0.09	1.07	0.90, 1.28
COVID onset	−0.80*	0.26	0.45	0.27, 0.75	−0.85*	0.26	0.43	0.26, 0.72	−0.82*	0.27	0.45	0.27, 0.75	−0.81*	0.27	0.44	0.26, 0.75
Tobacco use	0.03*	0.01	1.03	1.00 1.05	0.03*	0.01	1.03	1.00 1.05	0.03*	0.01	1.03	1.00 1.05	0.03*	0.01	1.03	1.00 1.05
Alcohol use	−0.01	0.02	0.99	0.96 1.03	−0.01	0.02	0.99	0.96 1.03	−0.01	0.02	0.99	0.96 1.03	−0.01	0.02	0.99	0.96 1.03
**Model fit**																
AIC	18859.526				20560.001				20872.686				20938.261			
BIC	19197.490				20952.885				21265.570				21331.145			

*Significant at the 0.05 level (2-tailed). SC, self-control; AIC, akaike information criterion; BIC, Bayesian information criterion. Model 1 shows the effects of planful self-control, threat, deprivation, contextual stress, and covariates on metabolic risk. Model 2 shows the effects of planful self-control, threat, deprivation, contextual stress, covariates, and the interaction between striving and deprivation on metabolic risk. Model 3 shows the effects of planful self-control, threat, deprivation, contextual stress, covariates, and the interaction between striving and threat on metabolic risk. Model 4 shows the effects of planful self-control, threat, deprivation, contextual stress, covariates, and the interaction between striving and contextual stress on metabolic risk.

## Discussion

Emerging research suggests that investigations of resilience among Black American men must content with the possibility that a resilience observed in psychosocial functioning may take a tool on health. The current study investigated the divergent effects of planful self-control on young adult, Black American men’s psychosocial well-being and metabolic risk. Specifically, we used a prospective design to (a) examine the direct relationship between Black American men’s planful self-control and specific psychosocial outcomes (e.g., educational attainment, job satisfaction, and depressive symptoms) and (b) examine the moderating effects of childhood and young adult adversities on the relationship between emerging adult Black American men’s planful self-control and metabolic risk. Study findings revealed that increased levels of planful self-control were directly associated with (a) lower levels of depressive symptoms, (b) higher levels of job satisfaction, (c) an increased likelihood of earning a bachelor’s degree, and (d) increased metabolic risk. Our findings indicated that within the context of high levels of childhood deprivation, increased levels of planful self-control were associated with elevated metabolic risk.

Planful self-control was directly associated with positive psychosocial outcomes and with increased metabolic risk. These findings are consistent with emerging research documenting the protective effects of planful self-control on psychosocial health and socioeconomic outcomes at the detriment of physiological health (Sellers and Neighbors, 2008; Chen et al., 2020). According to Brody et al. (2013), planful self-control can be emotionally and socioeconomically beneficial as men are able to develop competencies in the face of adversity, enabling them to *beat the odds*. However, prior evidence indicates that high-effort coping can be physiologically strenuous, which contributes to increased stress on the body that eventually results in poor physical health (Bennett et al., 2004). For example, Robinson and Thomas Tobin (2021) examination of the associations between high effort coping, and physical and mental health, among 627 Black Americans found that high effort coping was associated with decreases in depressive symptoms but increases in allostatic load (i.e., an indicator of physiological dysregulation that occurs as a response to stress). Our findings extend prior examinations of skin-deep resilience by demonstrating the direct benefits that planful self-control can have on young Black men’s outcomes related to depressive symptoms, job satisfaction, and educational attainment, while simultaneously demonstrating the direct detrimental effects that planful self-control can have on men’s cardiovascular health.

We hypothesized that the influence of planful self-control on metabolic risk would be particularly pronounced among men experiencing contextual adversity in their lives. A contextual stress by planful self-control interaction did not emerge. This is inconsistent with previous studies (Brody et al., 2013, 2020; Chen et al., 2020); however, these studies tended to be with younger samples and did not focus solely on men. It may be the case that skin deep resilience emerges earlier in development. We leveraged retrospective data on childhood adversity to consider if exposure to different childhood adversities would interact with planful self-control to predict increases in metabolic risk. Consistent with a skin-deep resilience pattern, we found that childhood deprivation interacted with planful self-control to predict metabolic risk, high levels of planful self-control combined with childhood deprivation was associated with increases in men’s metabolic risk. This pattern characterized about one-third of the sample. Our findings are consistent with prior developmental research that suggests that restricted access to resources in childhood can have enduring effects on physiological health (Brody et al., 2013). For example, Gaydosh et al.’s (2018) study examining the effects of childhood deprivation on the association between college attainment and metabolic risk demonstrated that within the context of high levels of childhood deprivation, college completion was associated with higher metabolic syndrome among young Black and Hispanic adults.

Our results indicate that Black men who have experienced childhood deprivation may experience a “zero-sum” game regarding planful self-control and their well-being. Should Black men choose to strive for success, they increase their risk of experiencing significant long-term physical health issues. Yet should Black men choose not to strive for success, they may then experience significant psychosocial challenges. These findings highlight the need for (a) further research documenting the complicated effects of skin-deep resilience and Black American men’s health, and (b) targeting childhood deprivation screening tools and interventions.

Inconsistent with hypotheses, childhood experiences of threat did not significantly influence the relationship between planful self-control and metabolic risk. The insignificance of this interaction may be related to the differential developmental effects of childhood threat, in comparison to childhood experiences of deprivation (Kinniburgh et al., 2005). For example, adults who experience significant threat during childhood may prioritize physical and emotional safety and security over their potential futures and adult socioeconomic stability (Edwards et al., 2021). Behaviorally this priority may be represented by men choosing to enter the workforce, often at dissatisfying jobs, instead of pursuing higher education to gain independence. In this instance, men’s focus may not be on upward mobility, or planful self-control, but instead on safety, and stabilization. Prior theorizing, coupled with our results, indicate that planful self-control and skin-deep resilience may be heavily influenced by men’s psychological desire for upward socioeconomic mobility due to their experiences of resource deprivation in childhood (Pitesa and Pillutla, 2019). In comparison, men’s psychological need for physical and emotional safety in adulthood following experience of abuse in childhood may not interact with planful self-control to compromise men’s physiological health.

Limitations to the study are notable. Findings may not generalize to women or to men from urban areas or of other racial groups. Information on planful self-control during childhood and adolescence and baseline information on metabolic risk, would allow better specification of when the skin-deep resilience pattern emerges. There are contextual factors that affect rural Black men’s health (e.g., community norms) that are not measured in this study. Study findings may not generalize to non-rural Black men or to Black women. The use of biomarker measurements allows us to investigate risk before disease onset when many conditions are asymptomatic or undetected via traditional clinical screening. Nevertheless, it remains unknown whether such risk will ultimately manifest in morbidities, or if high planful self-control individuals will be able to translate their accumulating advantage into better health as they age. RDS sampling may be subject to selection biases and self-report measures are subject to response bias. Documenting the health consequences associated with planful self-control in early adulthood provides a foundation from which to understand different aging trajectories among those from disadvantaged backgrounds.

## Data Availability Statement

The data supporting the conclusions of this article will be made available by the authors under a limited disclosure agreement. Requests to access the datasets can be directed to the corresponding author SK, smkogan@uga.edu.

## Ethics Statement

The studies involving human participants were reviewed and approved by University of Georgia Institutional Review Board. The patients/participants provided their written informed consent to participate in this study.

## Author Contributions

SK, AR, and MC wrote the first draft of the manuscript. AR conducted statistical analyses. CC, HZ, EK, and DA provided feedback on versions of the manuscript. All authors collaboratively conceptualized the study, developed hypotheses, interpreted study results, and approved the submitted version.

## Author Disclaimer

The content is solely the authors’ responsibility and does not necessarily represent the official views of the National Institute on Drug Abuse, the National Institute on Alcohol Abuse and Alcoholism, or the National Institutes of Health.

## Conflict of Interest

The authors declare that the research was conducted in the absence of any commercial or financial relationships that could be construed as a potential conflict of interest.

## Publisher’s Note

All claims expressed in this article are solely those of the authors and do not necessarily represent those of their affiliated organizations, or those of the publisher, the editors and the reviewers. Any product that may be evaluated in this article, or claim that may be made by its manufacturer, is not guaranteed or endorsed by the publisher.
