# Supplementary material for: Planful Self-Control, Metabolic Risk, and Psychosocial Outcomes Among Young, Black Men: A Test of Skin-Deep Resilience Theory
Source: Front Psychol. 2022 Jun 9;13:806955. doi: 10.3389/fpsyg.2022.806955 (PMC9218602; doi:10.3389/fpsyg.2022.806955)
Supplement: Supplementary file 1 [file Table_1.DOCX]

| **Supplement Table 1**  Correlations of Study Variables | | | | | | | | | | | | | | |  |  |
| --- | --- | --- | --- | --- | --- | --- | --- | --- | --- | --- | --- | --- | --- | --- | --- | --- |
|  | 1 | 2 | 3 | 4 | 5 | 6 | 7 | 8 | 9 | 10 | 11 | 12 | 13 | 14 | 15 | 16 |
| [1] Metabolic Risk | -- |  |  |  |  |  |  |  |  |  |  |  |  |  |  |  |
| [2] Planful Self-Control | 0.14^*^ | -- |  |  |  |  |  |  |  |  |  |  |  |  |  |  |
| [3] Childhood Threat | -0.07 | -0.11^*^ | -- |  |  |  |  |  |  |  |  |  |  |  |  |  |
| [4] Childhood Deprivation | -0.03 | -0.09^*^ | 0.38^**^ | -- |  |  |  |  |  |  |  |  |  |  |  |  |
| [5] Contextual Stress | -0.03 | -0.25^**^ | 0.25^**^ | 0.20^**^ | -- |  |  |  |  |  |  |  |  |  |  |  |
| [6] Depressive Symptoms | 0.03 | -0.18^**^ | 0.16^**^ | 0.16^**^ | 0.23^**^ | -- |  |  |  |  |  |  |  |  |  |  |
| [7] Job satisfaction | 0.01 | 0.29^**^ | -0.06 | -0.08 | -0.27^**^ | -0.25^**^ | -- |  |  |  |  |  |  |  |  |  |
| [8] Educational Attainment | 0.03 | 0.23^**^ | 0.02 | -0.11^*^ | -0.09 | -0.03 | 0.04 | -- |  |  |  |  |  |  |  |  |
| [9] Age in years | -0.04 | -0.13^*^ | -0.03 | -0.01 | 0.17^**^ | -0.04 | -0.06 | -0.08 | -- |  |  |  |  |  |  |  |
| [10] Healthy Diet | 0.04 | 0.22^**^ | -0.01 | -0.06 | -0.20^**^ | -0.01 | 0.07 | 0.22^**^ | 0.02 | -- |  |  |  |  |  |  |
| [11] Physical Exercise | 0.00 | 0.09 | -0.02 | 0.06 | 0.04 | -0.09 | 0.01 | -0.15^**^ | 0.03 | 0.03 | -- |  |  |  |  |  |
| [12] COVID Onset | -0.19^**^ | -0.07 | -0.01 | 0.01 | 0.03 | -0.03 | -0.02 | -0.07 | 0.48^**^ | 0.02 | -0.07 | -- |  |  |  |  |
| [13] Maternal Education | 0.01 | 0.01 | -.089^*^ | -0.04 | -0.01 | -0.06 | 0.06 | 0.13^*^ | 0.09 | 0.06 | 0.02 | 0.08 | -- |  |  |  |
| [14] Paternal Education | -0.05 | -0.02 | -0.04 | -0.02 | 0.00 | -0.04 | 0.04 | 0.09 | 0.09 | 0.04 | 0.03 | 0.07 | 0.97^**^ | -- |  |  |
| [15] Tobacco Use | 0.05 | -0.06 | 0.01 | 0.08 | 0.05 | 0.16** | -0.06 | -0.12* | -.09 | -0.10 | 0.01 | -0.01 | -0.04 | -0.24** | - |  |
| [16] Alcohol Use | -0.03 | -0.01 | 0.00 | -0.01 | 0.13* | 0.05 | 0.01 | 0.01 | -0.05 | -0.08 | 0.11* | 0.04 | -0.00 | -0.04 | 0.24** |  |
| *Note*. *. Correlation is significant at the 0.05 level (2-tailed). **. Correlation is significant at the 0.01 level (2-tailed). | | | | | | | | | | | | | | |  |  |
